# Association of early immune-related adverse events with treatment efficacy of neoadjuvant Toripalimab in resectable advanced non-small cell lung cancer

**DOI:** 10.3389/fonc.2023.1135140

**Published:** 2023-05-15

**Authors:** Ye Tao, Xiang Li, Bing Liu, Jia Wang, Chao Lv, Shaolei Li, Yuzhao Wang, Jinfeng Chen, Shi Yan, Nan Wu

**Affiliations:** Beijing Cancer Hospital, Peking University, Beijing, China

**Keywords:** immune-related adverse events, neoadjuvant, Toripalimab, non-small cell lung cancer, immunotherapy

## Abstract

**Background:**

Neoadjuvant immunotherapy with anti-PD-1 was proved promising in resectable non-small cell lung cancer (NSCLC). Immune-related adverse events (irAEs) have been preliminarily implicated their association with treatment efficacy. Here we elucidated the early onset of irAEs associated with better clinical outcomes in a prospective study (Renaissance study).

**Methods:**

We conducted the prospective study of NSCLC patients treated by neoadjuvant Toripalimab (240mg, every 3 weeks) plus double platinum-based chemotherapy from December 2020 to March 2022 at Peking University Cancer Hospital. Patients were enrolled if they have resectable IIB-IIIB NSCLC without EGFR/ALK mutation. Data were analyzed to explore the relationship between clinical outcome and irAEs after neoadjuvant treatment. A multidisciplinary team including physicians, surgeons, and radiologists, confirmed the irAEs according to the clinical manifestation. The relationship between irAEs and pathological outcomes was analyzed. The Renaissance study was approved by the Peking University Ethic board (2020YJZ58) and registered at https://clinicaltrials.gov/ as NCT04606303.

**Results:**

Fifty-five consecutive patients were enrolled with a male-to-female ratio of 10:1, the median age was 62 years old (IQR: 45-76), of which 44 patients (80%) were diagnosed with squamous cell carcinoma. Forty-eight of 55 patients finally received thoracic surgery with a median preoperative waiting time of 67 days (IQR 39-113 days). Pathological results demonstrated that 31 (64.6%) patients achieved major pathological response (MPR) and 24 (50.0%) achieved complete pathological response (pCR). Among 48 patients who received R0 resection, immunotherapy-related thyroid dysfunction, rash/pruritus and enteritis occurred in 11 patients (22.9%), 7 patients (14.6%), and 1 patient (2.1%), respectively. Six patients (54.5%) with thyroid dysfunction achieved MPR with 5 (45.5%) achieved pCR, and a median time to onset was 45 days (IQR 21-91 days). Six patients (85.7%) with rash or pruritus achieved MPR and 5 patients (71.4%) achieved pCR, with median time to onset being 8 days (IQR 6-29 days). Furthermore, irAEs had no significant influence on operation time (170.6 min vs 165.7 min, P=0.775), intraoperative blood loss (67.4 mL vs 64.3 mL, P=0.831) and preoperative waiting time (93 days vs 97 days, P=0.630) when comparing with patients without irAEs (**Figure 1**).

**Conclusion:**

The immunotherapy-related rash is potentially associated with pathological outcomes in NSCLC patients after neoadjuvant chemo-immunotherapy, suggesting easy-to-find irAEs, such as rash, can be used as indicators to predict response to neoadjuvant chemo-immunotherapy.

**Clinical trial registration**

clinicaltrials.gov/, identifier NCT04606303.

**Figure 1:**
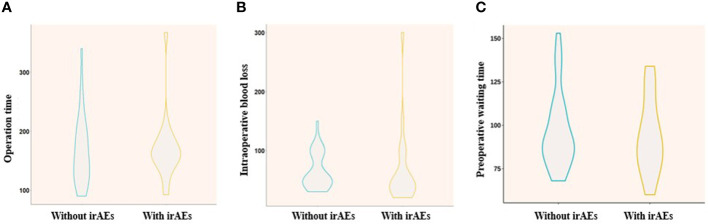
Comparison of operation time **(A)**, intraoperative blood loss **(B)**, and preoperative waiting time **(C)** between “with irAEs” and “without irAEs”.

## Introduction

Lung cancer counts as the leading cause of cancer-related death worldwide (1). Only one-quarter of patients with non–small-cell lung cancer (NSCLC) are diagnosed with early-stage disease and eligible for curative-intent surgery (2). The emergence of neoadjuvant therapy made it possible for locally advanced patients to accept surgical resection (3), and the addition of immunotherapy further improve the pathological outcomes of these patients in CheckMate 816 trial (4) and several single arm clinical trials compared with mono-chemotherapy (5–10). Immunotherapy, a significant treatment emerging in the past decade, has changed the paradigm of lung cancer treatment and brought great benefits to patients with PD-L1-expressing, locally advanced or metastatic NSCLC and locally advanced patients after radical resection or concurrent chemoradiotherapy (11–15).

As to neoadjuvant treatment, it was reported in the CheckMate-816 clinical trial that the median event-free survival was 31.6 months with nivolumab plus chemotherapy, and the percentage of patients with a pathological complete response (pCR) was 24.0% (4). Zhao et al. (9) found that Toripalimab, a PD-1 monoclonal antibody, plus platinum-based doublet chemotherapy yields a substantial major pathological response (MPR) rate (50% in the per-protocol population) with manageable toxicity and feasible resection in stage III NSCLC. Toripalimab combined with stereotactic body radiation therapy (SBRT) is also effective as a neoadjuvant regimen (16). There are also a larger number of clinical trials of neoadjuvant chemotherapy combined with immunotherapy (shown in the following chart). The pCR rates and treatment-related adverse events (TRAE) occurrence differ from 9% to 71% and 23% to 92.6%.

It is often observed in clinical practice that patients with immune-related adverse events (irAEs) may be more likely to achieve pCR after neoadjuvant immunotherapy. Some literature has pointed out that the irAEs happened due to the activation of T cells, which could help to kill the tumor cell. A retrospective study reported that patients with irAEs showed improved effectiveness over patients without irAEs (17, 18). To elucidate the association between the occurrence of irAEs and the pCR/MPR rate after R0 resection, data was analyzed among resectable locally advanced NSCLC patients to determine whether irAE could act as a prognostic factor to predict the efficacy of immunotherapy.

## Methods

### Patients

We enrolled patients with resectable stage IIB-IIIB NSCLC (according to the staging criteria of the American Joint Committee on Cancer, 8th edition) without previous anticancer therapy, an Eastern Cooperative Oncology Group performance-status score of 0 or 1 (on a 5-point scale in which higher scores reflect greater disability) without EGFR/ALK mutation. Patients had to have measurable disease according to the Response Evaluation Criteria in Solid Tumors, version 1.1, and pretreatment tumor tissue available to assess the expression of programmed death ligand 1 (PD-L1). Patients with known ALK translocations or EGFR mutations were excluded. The inclusion criteria were: 1) cytological or histological confirmation of NSCLC; 2) primary staged as clinical or pathological stage IIB-IIIB; 3) completed at least one cycle of immunotherapy. The exclusion criteria were 1) EGFR/ALK mutation, 2) important organ dysfunction before treatment; 3) did not complete a follow-up visit of the first cycle at the time of data collection; 4) previously accepted anti-tumor treatment.

### Trial design and treatment

In the prospective study, NSCLC patients were treated with neoadjuvant Toripalimab (240mg, every 3 weeks) plus double platinum-based chemotherapy from December 2020 to March 2022 at Peking University Cancer Hospital. Patients received cisplatin 75mg/m2 and pemetrexed 500 mg/m2 for adenocarcinoma, or cisplatin 75mg/m2 and nab-paclitaxel 260 mg/m2 for other subtypes on day 1-2 of each 21-day cycle. And intravenous Toripalimab was used on day 1 with chemotherapy of each 21-day cycle. Surgical resection was performed 6-8 weeks afterward. We analyzed the data of patients in the Renaissance study and explored the association between clinical outcomes and irAEs after the commencement of neoadjuvant Toripalimab treatment. The study was approved by the Peking University Ethic board (2020YJZ58) and registered at https://clinicaltrials.gov/ as NCT04606303. All patients signed written informed consent forms, and all data were deidentified.

### End points and assessments

All patients were staged according to the 8th Edition American Joint Committee on Cancer staging system. Characteristics of patients were summarized, including age, sex, pathological type, smoking history, tumor-node-metastasis (TNM) stage, immunotherapy-related adverse events (irAEs), and residual viable tumor (RVT).

The primary endpoints were major pathological response (MPR) rate (19) (≤10% residual viable tumor cells in the primary tumor and sampled lymph nodes). The second end points were the emergence of irAEs, pathological complete response (pCR) rate (0% residual viable tumor cells in the primary tumor and sampled lymph nodes), objective response rate, R0 resection rate, perioperative safety, and event-free survival.

### Definition

IrAEs were defined as having a potential immunological basis that required more frequent monitoring and potential intervention assessed by a multidisciplinary team of physicians, surgeons, and radiologists. IrAEs were classified according to the National Cancer Institute Common Terminology Criteria for Adverse Events (NCI-CTCAE) version 4.03 (20) and were handled according to the guidelines on the management of immunotherapy-related toxicities (21, 22). The time to onset of irAE was defined as the time from the start of immunotherapy to the occurrence of irAE. Event-free survival (EFS) was defined as the time from the start of immunotherapy to any progression of disease precluding surgery, progression or recurrence of disease after surgery, progression of disease in the absence of surgery, or death from any cause.

### Statistical analysis

Descriptive statistics of baseline, clinicopathological, and operation-related characteristics were listed. The pCR, MPR, R0 resection rate, the occurrence of irAEs, the time interval between Toripa, limab use and the occurrence of irAE, and event-free survival were calculated. SPSS 26.0 (IBM, Armonk, NY, USA) was used for statistical analysis. The continuous data were presented as medians (ranges) and analyzed using the Mann-Whitney U-test. The categorical data were presented as numbers (percentages) and analyzed using the chi-square or Fisher’s exact test. And the survival outcome data were presented as average (ranges) and analyzed by the Cox proportion risk regression model. The Kaplan-Meier curve was analyzed by Log-Rank analysis. Reported P values are two-sided, and the significance level was set at 0.05 for all analyses unless otherwise noted.

## Results

### Patient characteristics

**Figure 2:**
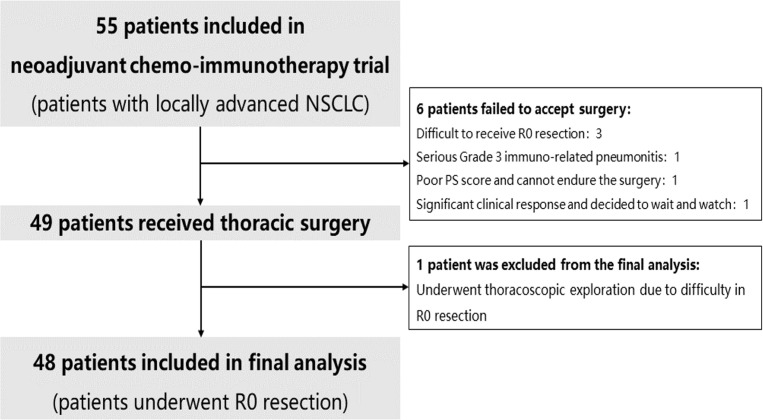
Flow chart of including patients.

From December 2020 to March 2022, 55 patients met the inclusion criteria, the median age was 62 years old (IQR 55-66 years old) (**Figure 2**). Overall, 50 patients (90.9%) were men, 49 patients (89.1%) had a smoking history, 16 patients (29.1%) were stage IIB, 31 patients (56.4%) were stage IIIA, 8 patients (14.5%) were stage IIIB. Nine patients (16.4%) were staged as N0, 16 patients (29.1%) were staged as N1, and 30 patients (54.5%) were staged as N2. Forty-four patients were diagnosed with squamous cell carcinoma (80%), nine patients were diagnosed with adenocarcinoma (16.4%), and 2 patients were proven to have adenosquamous lung cancer.

### Treatments and TRAE outcomes, especially irAEs outcomes

**Figure 3:**
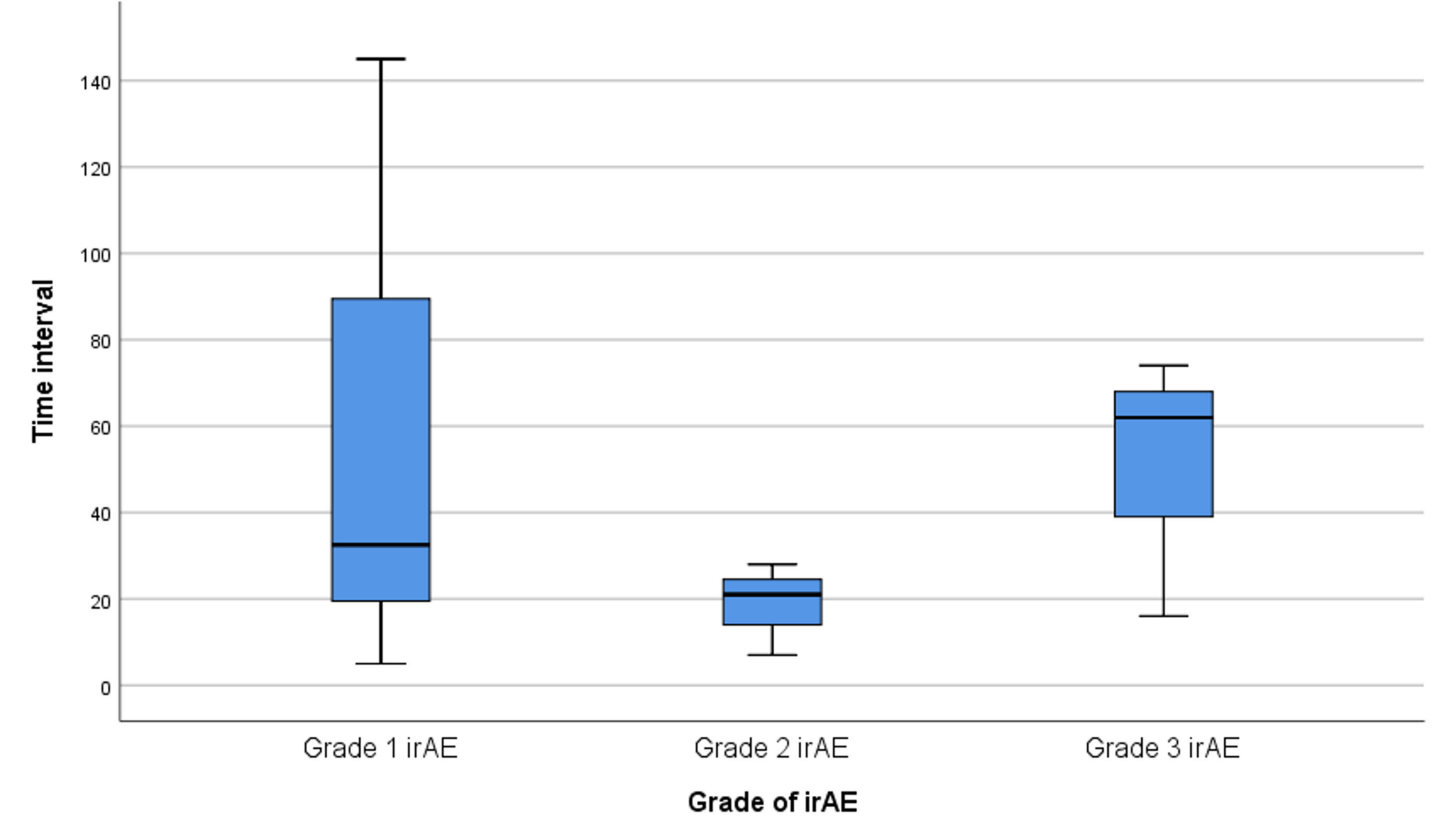
Comparison of the time interval between commencement of Torapalimab and diagnosis of different grades of irAEs. irAEs refers to immunotherapy-related adverse events.

About 69.1% of patients accepted 2 cycles of neoadjuvant Toripalimab plus chemotherapy, 14 patients accepted 3 cycles of neoadjuvant Toripalimab plus chemotherapy (25.5%), one patient received 4 cycles of neoadjuvant Toripalimab plus chemotherapy (1.8%), and 2 patient received one cycle of neoadjuvant Toripalimab plus chemotherapy (3.6%) (**Table 1**) One of the 2 patients that only take 1 cycle was due to bile tract infection, another patient suffered severe immune-related enteritis and can’t continue immunotherapy. TRAE occurred in 53 patients (96.4%), which contains myelosuppression, vomiting, nausea, alopecia, acroanesthesia, anorexia, hepatic function impairment, etc. Among them, 17 patients (30.9%) suffered Grade 3-4 TRAE. While there were 22 patients (40%) involved in this clinical trial were diagnosed with irAEs. Immune-related thyroid dysfunction, rash/pruritus, enteritis, and pneumonitis occurred in 12 patients (21.8%), 7 patients (12.7%), 2 patients (3.6%), and 1 patient (1.8%), respectively. Among these 22 patients, 6 patients suffered Grade 2-3 irAEs. The common Grade 2-3 irAEs were rash and pruritus (9.1%), immune-related enteritis (9.1%), immune-related pneumonitis (4.5%), and thyroid dysfunction (4.5%) (**Table 2**). The median time between the commencement of Torapalimab and diagnosis of irAEs was 28.5 days (IQR: 18.3-77.5 days). And the median time interval between the commencement of Torapalimab and diagnosis of grade 1, grade 2, and grade 3 irAEs were 32.5 days,21 days, and 62 days. Grade 1-2 irAEs appear to occur earlier than Grade 3 (**Figure 3**). Grade 1-2 rash was usually treated withsteroids ointment and oral antihistamines. Grade 1-2 thyroid dysfunction was treated with thyroid hormone supplements. While immune-related pneumonitis and enteritis were treated with intravenous steroids injection and sequentially dose decreased till suspension, symptomatic treatment was also used when patients suffered serious dyspnea or diarrhea. In our research, two patients with grade 2 irAEs (9.1%) and 3 patients with grade 3 irAEs (13.6%) were treated with glucocorticoid therapy. The median glucocorticoid-using time duration was 12 days (IQR: 7-79 days).

**Table 1:** Characteristics of included patients.

	N (%)
Age (IQR)	61.5 (55-66)
Gender	
male	50 (90.9%)
female	5 (9.1%)
Smoking history	
Yes	49 (89.1%)
No	6 (10.9%)
Staging	
IIB	16 (29.1%)
IIIA	31 (56.4%)
IIIB	8 (14.5%)
Staging of Lymph nodes	
N0	9 (16.4%)
N1	16 (29.1%)
N2	30 (54.5%)
Pathology	
Squamous cell carcinoma	44 (80.0%)
Adenocarcinoma	9 (16.4%)
Adeno-squamous carcinoma	2 (3.6%)
Cycles of Neoadjuvant Therapy	
1	2 (3.6%)
2	38 (69.1%)
3	14 (25.5%)
4	1 (1.8%)

**Table 2:** irAE of all 55 patients involved in trial.

Type of irAEs	Any Grade	Grade 2-3	Median Time Interval (Days)
Hyperthyroidism	12	1 (4.5%)	45.5(IQR 21-96.3)
Rash**/**pruritus	7	2 (9.1%)	8(IQR 6-29)
Enteritis	2	2 (9.1%)	45
Pneumonitis	1	1 (4.5%)	62
Total	22	6 (27.3%)	28.5 (IQR 18.3-77.5)

### Patients received surgery

Forty-eight of 55 patients finally received thoracic surgery with a median time interval of 67 days (IQR 39-113 days) after neoadjuvant treatment. Pathology confirmed that 31 (64.6%) patients achieved MPR and 24 (50.0%) patients achieved pCR.

In a total of 48 patients who received R0 resection, eleven patients suffered from thyroid dysfunction, of which six patients achieved MPR and 5 patients achieved pCR, the median time from treatment initiation to onset was 45 days (IQR 21-91 days). Six patients with rash or pruritus achieved MPR, and 5 patients achieved pCR, the median time to onset was 8 days (IQR 6-29 days). One patient with enteritis achieved pCR, and the time interval between treatment and the onset of enteritis was 16 days. Among these patients with irAEs, three patients suffered grade 2-3 irAEs and achieved pCR(**Table 3**).

**Table 3:** irAE of 48 patients received R0 resection.

Type of irAEs	Any Grade	Grade 2-3	Median Time Interval (IQR)	pCR	pCR rate	MPR	MPR rate
Hyperthyroidism	11	0	45 days(21-91 days)	5	45.5%	6	54.5%
Rash**/**pruritus	7	2	8 days(6-29 days)	5	71.4%	6	85.7%
enteritis	1	1	16 days	1	100%	1	100%
pneumonitis	0	0	0	0	0	0	0
Total	19	3	22 days (16-46 days)	11	57.9%	13	68.4%

Furthermore, as to the safety of thoracic surgery after neoadjuvant Toripalimab plus chemotherapy, irAEs showed no significant influence on the operation time (170.6 min vs 165.7 min, *p*=0.775) (**Figure 1A**), intraoperative blood loss (67.4 mL vs 64.3 mL, *p*=0.831) (**Figure 1B**) and preoperative waiting time (93 days vs 97 days, *p*=0.630) (**Figure 1C**). The pathological response outcomes of different stage of NSCLC was presented in **Table 4**. It showed that Stage IIB to IIIA might benefit mostly from neoadjuvant immunochemotherapy, and no significant difference was found between clinical stage and pathological response (*p*=0.27).

**Table 4:** The results of pathological response for different clinical stage of NSCLC.

Clinical Stage /pathological response	IIB	IIIA	IIIB	Total
pCR	7	16	1	24
MPR not pCR	2	4	1	7
Non-MPR	4	8	5	17

**Figure 4:**
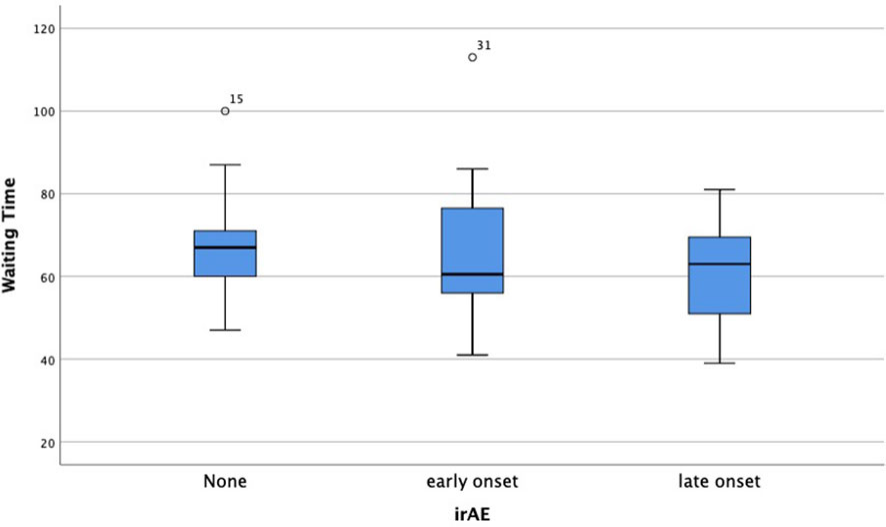
Comparison of waiting time after the last cycle of immunotherapy between early, late-onset or no occurrence of irAEs.

We also analyzed the time-onset of irAE and the time interval between the last cycle of neoadjuvant therapy and surgery (**Figure 4**), the *p*-value didn’t show a significant difference, which further confirms that the occurrence of irAE would not delay surgery and neoadjuvant immuno-chemotherapy could be estimated as dependable and safe.

### Patients failed to receive surgery

**Figure 5:**
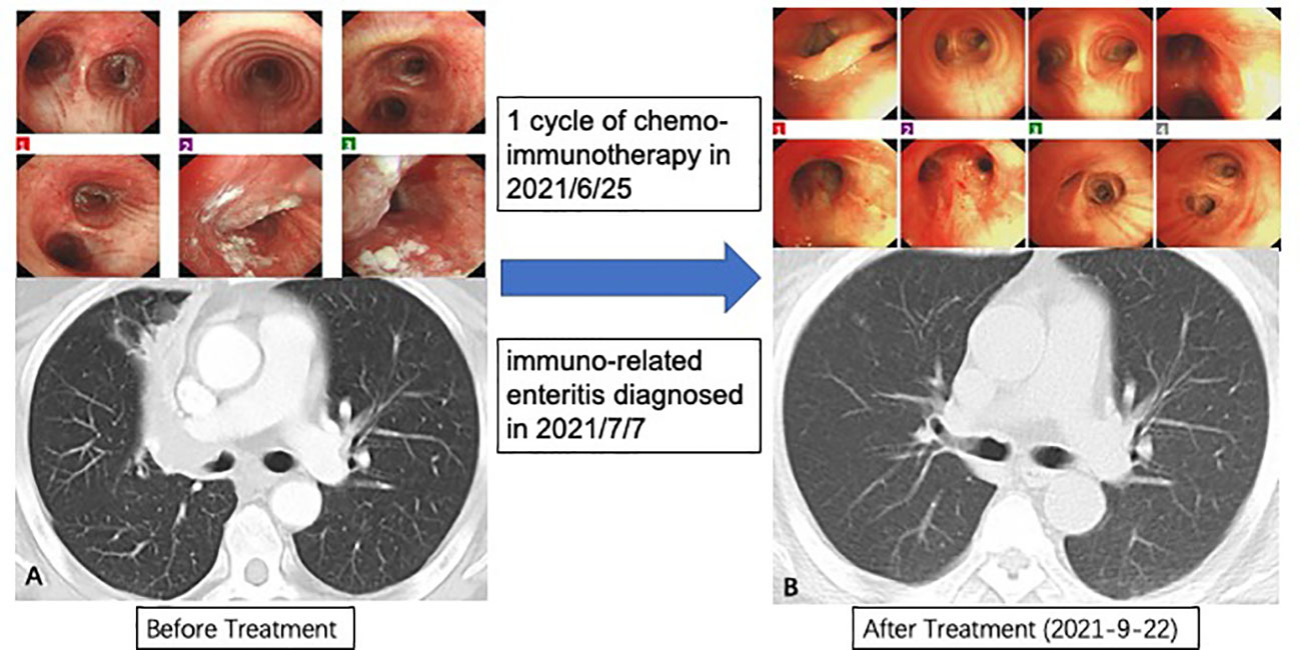
Procedures of treatment and response. **(A)** Baseline characteristic of lungs; **(B)** Great clinical response in bronchoscopy and computed tomography.

There is one patient who decided to watch and wait after complete clinical response (CCR) (**Figure 5**). This patient was diagnosed with lung adenocarcinoma, cT2N2M0, stage IIIA. His CT examination in May 2021 revealed truncated bronchial stenosis in the upper lobe of the right lung, irregular soft tissue foci next to the hilum, about 32*22mm, with distal lung tissue atelectasis. There were also multiple enlarged lymph nodes in the mediastinum and the right hilum. Bronchoscopy showed a bulge around 2/3 circumference of the right main bronchus, with the upper edge about 5mm from the bulge. Pathological biopsy suggested moderately differentiated squamous carcinoma. He was treated with 1 cycle of ABP (albumin- bounded paclitaxel) + cisplatin + Toripalimab in 2021/6/25. The next cycle of treatment was canceled due to the development of severe immune-related enteritis which was treated at Peking Union Medical College Hospital subsequently. On 2021-9-22 his chest CT showed: the soft tissue masses near the right hilum disappeared. Multiple enlarged lymph nodes were reduced in size. PET-CT of the right pulmonary hilum did not show hypermetabolism. In 2021-12-16, he reviewed bronchoscopy and the biopsy reported mild chronic inflammation of the mucosa of the pseudostratified ciliated columnar epithelium, with two heterogeneous cells visible in the interstitium, considered to be cancer. In 2022-4-13, we reviewed the bronchoscopy again, and the biopsy showed no definite clue of lung cancer. In 2022-12-16, we retested the chest CT: it showed that the soft tissue thickening in upper lobe of the right lung near the hilum was as before, either was the bronchial stenosis in the right upper lobe.

**Figure 6:**
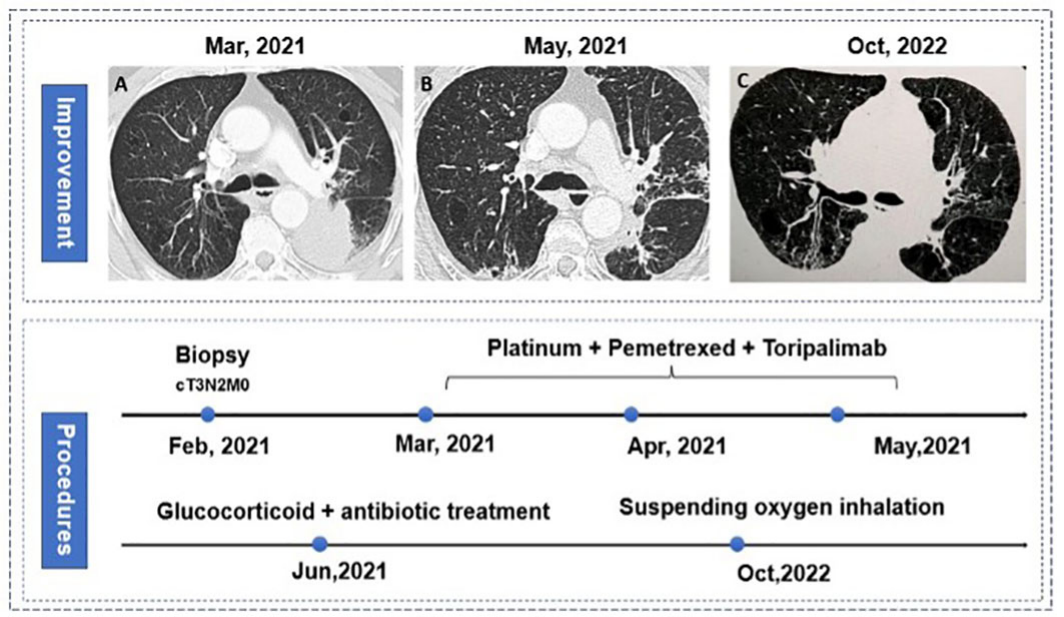
Procedures of treatment and pneumonitis. **(A)** baseline characteristic of lungs; **(B)** immune-related pneumonitis **(C)** great improvement in tumor and pneumonitis in bilateral lungs.

There were also 6 other patients who failed to receive surgery, of which three patients failed to receive surgery for the poor performance in radiological imaging, as well as 2 patients’ surgery were canceled respectively for the anatomical adhesion during surgical exploration and poor physical performance. Notably, one patient’s surgery was canceled for grade 3 immuno-related pneumonitis. This patient has diagnosed with stage IIIB adenocarcinoma without ALK translocation and EGFR mutation in the left lower lobe (**Figure 6A**), thus three cycles of neoadjuvant Toripalimab combined with chemotherapy were used for the surgical opportunity. While reduced exercise tolerance come out after the third cycle, he simultaneously had intermittent fever during hospitalization, chest computed tomography showed large-area ground-glass opacity with consolidation (**Figure 6B**), which confirmed the diagnosis of immuno-related pneumonitis. Arterial blood gas analysis showed an arterial partial pressure of oxygen of 68.2 mmHg. After standard treatment of glucocorticoid therapy combined with continued antibiotic treatment, the patient recovered from the server pneumonitis with continuous oxygen therapy. The patients achieved great improvement for 18 months from the treatment start (**Figure 6C**). And recently computed tomography showed that the tumor completely disappeared.

### Patients underwent adjuvant therapy

There are 14 patients who took adjuvant therapy, which includes chemo-immunotherapy with/without sequential immunotherapy for maintenance treatment, or directly adjuvant immunotherapy. There are 34 patients who didn’t take adjuvant therapy, the result of adjuvant therapy were presented in **Table 5**.

**Table 5:** The results of the of adjuvant therapy of 48 patients.

Pathological response /Adjuvant Therapy	Chemo-immunotherapy	Immunotherapy	None
pCR	1	2	21
MPR not pCR	2	1	4
Non-MPR	5	3	9
Total	8	6	34

### Survival analysis

The average EFS of patients without irAE (33 patients) was 22.7 months (95%CI 21.3-24.2 months). The median EFS of patients with irAE (22 patients) was 23.5 months (95%CI 21.0-25.9 months), and both median times were not reached, The log-rank analysis showed that the *p*-value was 0.61.

6 patients arrived at the end point of our study, 3 of them had encountered local recurrence, 2 of them had been diagnosed with metastatic lung cancer, and only 1 patient died during our study.

**Figure 7:**
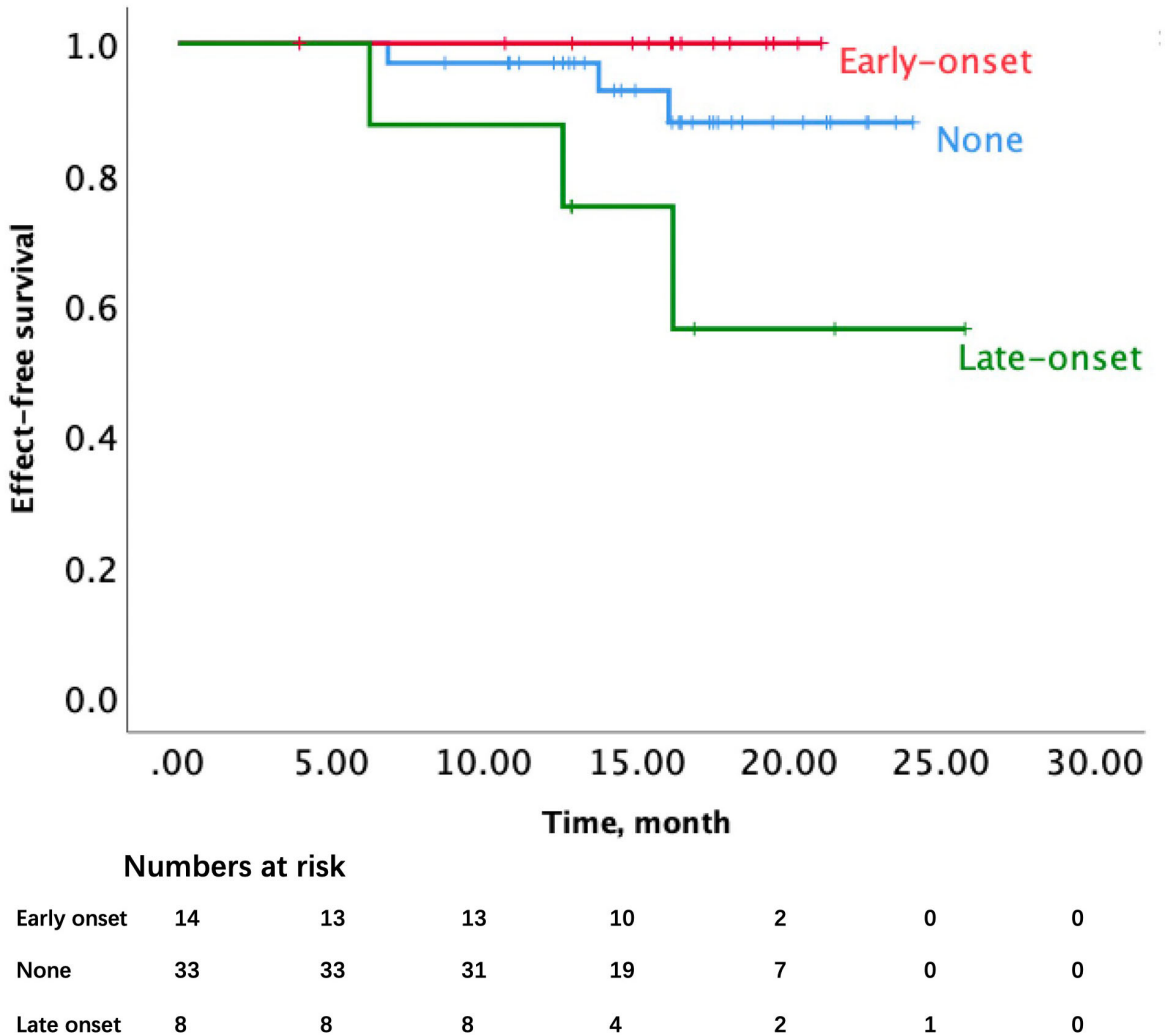
Event-free survival by early or late onset of irAE in all 55 patients.

We separated the patients with irAE into the early-onset and late-onset, each separately included 14 and 8 patients. The watershed between them was 3 months. Due to the small sample size, the HR of early-onset irAE had no significant difference, and the HR of late-onset irAE was 4.53 (*p*=0.065, 95%CI 0.91-22.5). While the log-rank analysis of the K-M curve showed a significant difference (*p*=0.021) (**Figure 7**).

## Discussion

Immunotherapy, targeting T-cell regulatory pathways, is widespreadingly recommended as first-line therapy for advanced lung cancer, which has been heralded as a promising treatment when combined with chemotherapy. Several international clinical trials provided the evidence that neoadjuvant immunotherapy plus chemotherapy performed well with higher rates of pathological response and controllable side effects (**Table 6**).

**Table 6:** The results of the terminated neoadjuvant immunotherapy trials for resectable NSCLC.

Trial	Identifier	Phase	Stage	Sample Size	Primary Endpoint	Treatment	R0 Rate	pCR	TRAE	MPR
LCMC	NCT02927301	II	IB–IIIB	181	MPR	atezolizumab monotherapy	76%	NA	41%	20%
Long et al.	NCT04304248	II	III	33	MPR	toripalimab	96.7%	45.5%	NA	60.6%
CheckMate159	NCT02259621	II	I-IIIA	22	Safety	Nivo	91%	15%	23%	45%
NADIM	NCT03081689	II	IIIA	46	PFS	Nivo	89%	71%	93%	85%
SAKK 16/14	NCT02572843.	II	IIIA(N2)	68	MPR	Durvalumab	93%	18%	88%	62%
J.Lei et al. (23)	NCT04338620.	II	IIIA, IIIB-N2	14	MPR	camrelizumab	50%	57.1%	NA	85.9%
Gao et al.	ChiCTR-OIC-17013726	1b	IA-IIIB	40	MPR	Sintilimab	92.5%	16.2%	52.5%	40.5%
H.Duan et al. (24)	–	II	IIA-IIIB	20	ORR	PD-1 inhibitors	95%	30%	NA	50%
Checkmate816	NCT02998528	III	IB-IIIA	358	EFS/pCR	Nivolumab	83.2%	24%	92.6%	NA
A.Tfayli et al. (25)	NCT03480230	II	IB-IIIA	15	ORR	Avelumab	73%	9%	NA	27%

According to the NADIM study, the MPR rate of all patients involved was 45% (7). As the CheckMate 816 trial found out: the pCR rate of Stage IIIA NSCLC was 23%, and the pCR rate of squamous cell carcinoma was slightly higher than adenocarcinoma, without significant difference (4).

Moreover, a large number of patients with NSCLC in China were diagnosed with non-squamous carcinoma (26). Epidermal growth factor receptor (EGFR) mutation is currently the most common target; EGFR mutations were more common in Chinese patients than in American patients (27), which might result in our patients that accepted immunotherapy of non-squamous NSCLC (11/55, 20%) is less than of squamous cell carcinoma (44/55, 80%). As a single-center study, our patients cannot present for the whole population in Beijing or China, which contributes to the inevitable selection bias of our study.

Previous articles reported that immunochemotherapy obtained shorter operative time, a higher rate of en bloc resection, and minimal invasion (4). As validated in our study, patients with irAE showed similar preoperative waiting time, operation time, and intraoperative blood loss, confirming its safety and reliability of neoadjuvant immunochemotherapy. The relationship between the irAEs and pathological response was explored as the predictor for the combination treatment. Hyperthyroidism, rash, and pruritus were the common adverse events reported in recent articles on immunochemotherapy. Intriguingly, rash and pruritus always come out early, and most of these patients were proven pCR finally. Moreover, some literature did confirm that irAE is connected with better OS in melanoma (28), non-squamous NSCLC (29) and ESCC (30). The specific types of irAE might predict better prognosis as well, such as colitis and diarrhea (31). Our initial idea was to find the association between the occurrence of specific irAE with a better pathological response. Unfortunately, no significant P value was found between irAE and pathological response. It had been reported that 3 months could serve as a watershed to identify the early-onset or late-onset irAEs (32), as we estimated, the median time between the commencement of Toripalimab and surgery was nearly 3 months, either. Thus we did a survival analysis of the onset of irAE and event-free survival and got a positive outcome, which might proclaim the early-onset irAE as a biomarker of better outcomes among locally advanced NSCLC patients with neoadjuvant immune-chemotherapy modality. Early-onset irAEs were mainly composed of rash/pruritus and thyroid dysfunction, both were easily monitored and handled during the preoperative treatment period. Patients with early-onset irAEs including rash or pruritus in neoadjuvant immunotherapy were more likely to achieve better event-free survival compared to late-onset irAEs. We only came out with this conclusion based on our 55-patient retrospective trial in our hospital, more rigorous randomized clinical trials are needed to find the association between them for neoadjuvant immunotherapy in locally advanced NSCLC.

To our best knowledge, immune checkpoint inhibitors are designed to attack malignancies by targeting the ligands, leading to T-cell activation for the attack against malignant cells. These ligands included cytotoxic T-lymphocyte antigen-4 (CTLA-4), programmed death protein 1 (PD-1), and programmed death ligand-1 (PD-L1). These corresponding medications upregulate the immune system and cause irAEs (33). Some adverse events may be mild, 14 patients with lung cancer who received immunotherapy reported that their hair turned black, and another case reported that one patient with metastatic cutaneous melanoma developed eyelash poliosis after undergoing treatment with combination immunotherapy with ipilimumab and nivolumab (34). In a responder analysis, an increase in overall survival was seen in patients with the related adverse event of special interest (AESI) compared with those with no related AESIs. About 57% of responding patients with a related AESI reported the AESI before documentation of response (35). Patients with rash/pruritus always had a higher proportion of major pathological responses. Researchers found that T cells that reacted to antigens shared in NSCLC lesions and the skin-mediated autoimmune skin toxic effects. These T cells may also have mediated the tumor regression in patients who responded to therapy (17). Compared with immune-related pneumonitis, rash/pruritus happened earlier and was easier to be detected, which may lessly hinder thoracic surgery for its stable nature. Thus, more active surgical treatment should be adopted when confronted with certain irAE such as rash/pruritus.

Immunotherapy efficacy predictors were explored recently; serum biomarkers or others were regarded as powerful predictors. For example, phenotype markers were explored in an end-staged solid tumor, like PD-L1 expression, TILs (36), or LAG3 (37), etc. And there are also genomic markers such as MSI-H, TMB (38), specific gene mutation (39), and ctDNA. Inflammatory biomarkers are also explored to predict the efficacy of neoadjuvant immunotherapy (40). However, there was still lacking authoritative clinical evidence for these predictors. Based on previous studies, ctDNA could count as promising and dependable marker of neoadjuvant immune-checkpoint inhibitors used in resectable locally advanced NSCLC (4, 41, 42). But the cost and fundamental establishments made it less useful in our clinical practice. Other biomarkers like TMB, MIS-H or PD-L1 expression couldn’t effectively predict the outcome of neoadjuvant immunotherapy (5, 7). As the pooled analysis published in February 2023 on JAMA Oncology found out, irAE did bring out better OS and PFS in Stage IV non-squamous NSCLC patients who underwent Atezo treatment (29). Our study also found that early-onset irAEs were associated with better event-free survival in resectable NSCLC patients who underwent neoadjuvant Toripalimab treatment.

Nevertheless, some severe irAE like immune-related pneumonitis or enteritis could stop patients from getting regular preoperative treatment or even surgery. Early-onset irAEs were mainly composed of rash/pruritus and thyroid dysfunction, both were easily monitored and handled during the preoperative treatment period, which also highly alleviated the anxiety of these patients. Thus, we finally came out with this conclusion: Easy-to-find irAEs, such as rash, can be used as indicators to predict response to neoadjuvant chemo-immunotherapy.

According to the other accomplished clinical trials of neoadjuvant immunotherapy, TRAE happened in 23%-93% of patients, but they barely mentioned specifically about irAEs. One literature reported the occurrence rate of irAE in the atezo-containing arm in Stage IV non-squamous NSCLC as 48% (29). The reported incidence of any-grade irAEs associated with ICI treatment ranges widely across agents and trials, from approximately 15% to 90% (43, 44).

A meta-analysis studied by De Velasco et al. reported the incidence of the most common irAEs of 21 randomized phase II/III trials from 1996 to 2016, which included a total of 6528 patients who received monotherapy (45). Within this cohort, across all ICIs, incidence of all-grade irAE was 30.4%, and the incidence of grade 3/4 events was 1.5% for colitis, 1.5% for liver toxicity, 1.1% for rash, 0.3% for hypothyroidism, and 1.1% for pneumonitis, added up to 5.8% grade3/4 occurrence.

The present study has several limitations. Firstly, this was a single-center study, not a randomized controlled trial, and the results were subjective to the inherent shortcomings of the single-center study. Patients in preparation for lung cancer surgery were enrolled, which may introduce a selection bias. Additionally, the difference was not significant perhaps due to the small sample size, the confounding effects of limited sample size cannot be ruled out. Secondly, different PD-1 inhibitors might result in different irAEs and lead to different efficiency, only Toripalimab was used in this cohort. Our study only included the use of Toripalimab, different kinds of immune-checkpoint inhibitors might result in the different modalities of irAEs. Thus, the results may not be proper for other PD-1 inhibitors, which need more evidence from randomized controlled trials. Finally, all the irAEs were assessed by investors in our hospital, limited by a paucity of irAEs evaluation criteria, which may cause subjective judgment bias.

In conclusion, some irAEs may point to the positive treatment effect, which may not be affected by the treatment cycles. The importance of accurately identifying irAEs that may benefit from neoadjuvant immunotherapy is critical, given the potentially positive indicator on treatment‐associated results.

## Data availability statement

The original contributions presented in the study are included in the article/supplementary material. Further inquiries can be directed to the corresponding authors.

## Ethics statement

The studies involving human participants were reviewed and approved by Peking University Ethic board (2020YJZ58). Written informed consent to participate in this study was provided by the participants’ legal guardian/next of kin. Written informed consent was obtained from the individual(s) for the publication of any potentially identifiable images or data included in this article.

## Author contributions

NW and SY supervised the study. NW, SY, YT, and XL conceived of and designed the study. YT and XL did the statistical analysis and wrote the drafted report. NW, SY, YT, XL, YW, and JC critically revised the manuscript. BL, JW, and CL organized and screened patients. All authors had access to all the raw datasets. All authors contributed to the article and approved the submitted version.
